# Persistent Borna Disease Virus (BDV) infection activates microglia prior to a detectable loss of granule cells in the hippocampus

**DOI:** 10.1186/1742-2094-5-16

**Published:** 2008-05-19

**Authors:** Mikhail V Ovanesov, Krisztina Moldovan, Kimberly Smith, Michael W Vogel, Mikhail V Pletnikov

**Affiliations:** 1 Department of Psychiatry and Behavioral Sciences, Johns Hopkins University School of Medicine, Baltimore, MD, USA; 2 Maryland Psychiatric Research Center, University of Maryland, Catonsville, MD, USA

## Abstract

Neonatal Borna Disease Virus (BDV) infection in rats leads to a neuronal loss in the cortex, hippocampus and cerebellum. Since BDV is a non-lytic infection *in vitro*, it has been suggested that activated microglia could contribute to neuronal damage. It is also conceivable that BDV-induced cell death triggers activation of microglia to remove cell debris. Although an overall temporal association between neuronal loss and microgliosis has been demonstrated in BDV-infected rats, it remains unclear if microgliosis precedes or results from neuronal damage. We investigated the timing of microglia activation and neuronal elimination in the dentate gyrus (DG) of the hippocampus. We found a significant increase in the number of ED1+ microglia cells as early as 10 days post infection (dpi) while a detectable loss of granule cells of the DG was not seen until 30 dpi. The data demonstrate for the first time that a non-lytic persistent virus infection of neurons activates microglia long before any measurable neuronal loss.

## Findings

Activation of microglia is a hallmark of many neurodegenerative conditions produced by genetic mutations or environmental factors, including viral infections [1,2]. However, whether microglia activation is a cause or consequence of neuronal loss often remains unclear. Neonatal Borna Disease Virus (BDV) infection in rats is an example of neurodegenerative behavioral disease that is associated with activation of microglia and astrocytes and loss of neurons in the DG of the hippocampus, cortex, and cerebellum [3-6]. While an overall temporal association of neuronal death with microgliosis has been shown in neonatally BDV-infected rats, the precise timing of microglia activation has not been studied, and it remains unknown if microglia activation precedes neuronal loss or results from it [7-9]. This question is of importance since BDV is a non-lytic neurotropic virus and does not kill neurons *in vitro*, suggesting that activation of microglia may trigger neuronal damage and/or exacerbate initial subtle dysfunctions produced by the virus. In this regard, BDV infection pathogenesis may resemble some other chronic neurodegenerative conditions where initial neuronal disturbances lead to activation of microglia and ensuing neurodegeneration [1,2]. Thus, we quantitatively evaluated the timing of microglia activation and neuronal injury at early time points post infection.

Pregnant Fischer344 rats at 16–18 days of gestation were purchased for the present study (Harlan, Indianapolis, IN). All rat pups were born in the animal facility at Johns Hopkins University School of Medicine, Baltimore, MD. Mothers and their litters were housed in 45 × 26 × 23 cm pan-type polypropylene cages with paper-chip bedding and an overhead wire grid supporting food pellets and a water bottle. The cages containing BDV and sham-infected animals were kept in a DUO-FLO biosafety cabinet (Bio-Clean Lab Product Inc., NJ). Rats of all the groups were maintained on a 12/12-hr light/dark cycle (lights on at 8 a.m.). Room temperature was maintained at approximately 21°C. The pregnant and nursing rats had free access to food and water. After weaning, on postnatal day 23, the rats continued to have free access to food and water. Rat pups were inoculated intracranially under hypothermia anesthesia with 26-G needles within 24 hours of birth either with 20μL of infectious inoculum containing 10^4 ^TICD50 of He-80 BDV strain or uninfected inoculum as described previously [3,10]. All experiments were performed with adherence to the National Institutes of Health guidelines on the use of experimental animals and the animal protocol approved by the Johns Hopkins Medical Institutions' Animal Care and Use Committee.

To investigate a precise temporal relationship between microglia activation and neurodegeneration, infected and uninfected rat pups were sacrificed at 8, 10, 17, and 30 days post infection (dpi) with EUTHASOL (Diamond Animal Health, Inc. IA) and immediately perfused with ice-cold phosphate buffered saline (PBS, pH = 7.4) followed by 4% paraformaldehyde. Brains were removed, postfixed for 4 hours, cryoprotected in 30% sucrose in PBS, cut sagittally in half and frozen on dry ice. The right and left halves were cut saggitally in 40-μm sections for either immunofluorescence or immunohistochemical staining using standard protocols. Brains sections were co-stained with rabbit anti-GFAP (1:800, Chemicon, Temecula, CA) as a marker for astrocytes; anti-ED1 MAB (1:100, Chemicon), anti-CD11b MAB (1:100, R&D), anti-Iba1 (1:800) as the markers for microglia, and anti-BDV N Mo18 MAB (1:100, a generous gift by Dr Juergen Richt, National Animal Disease Center, 2300 Dayton Avenue, Ames, IA) [11] antibodies followed by either FITC-conjugated or Cy3-conjugated secondary antibodies (1:200, Chemicon) or biotinylated anti-mouse (for anti-BDV) or anti-rabbit (for anti-GFAP) antibodies followed by DAB (a brown reaction product) or Vector^® ^VIP substrate (a purple reaction product) chemical visualization, respectively (Vector Lab, Burlingame, CA). Antibodies against ED1, CD11b and Iba1 were used to detect microglia [7,9]. Although monocytes, B and T cells, and macrophages can also express these markers, we refer to the labeled cells as microglia because mononuclear infiltration of the brain parenchyma during neonatal BDV infection in rats is minimal and transient [9]. In addition, a double staining with anti CD3 antibody was used to stain macrophages that were found only in the meninges. Those round macrophages were double positive for CD3 and ED1 but were morphologically different from stellar-shaped microglia in the hippocampal area (data not shown).

**Figure 1:**
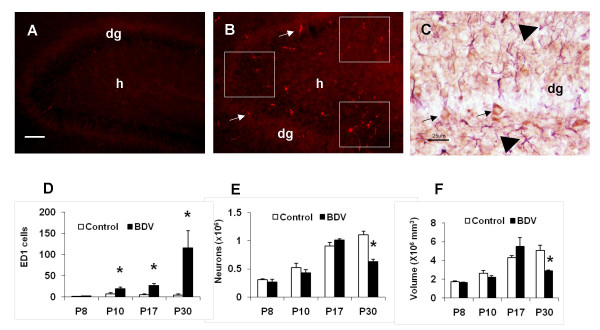
**Activation of microglia by BDV infection prior to neuronal loss in the hippocampus.** (A) No ED1+ cells in the section from a mock-infected rat, 10 dpi. Scale bar – 35 um; dg – the dentate gyrus; h – the hilus area. (B) BDV infection produces a significant increase in the number of ED1+ cells in the dentate gyrus. The white squares depict the areas where ED+ cells were counted; dg – the dentate gyrus; h – the hilus area; arrows point to ED1+ activated microglia cells; 10 dpi. (C) BDV-infection in neurons of the hippocampus. Double-immunostaining of the brain section from a BDV-infected rat at 10 dpi. Note BDV-positive (brown) staining in neurons (arrows) and GFAP-positive (purple) staining in astrocytes (arrowheads). Scale bar – 25 μm; dg – the dentate gyrus. (D) The quantitative analysis of ED1+ cells in the dentate gyrus of the hippocampus in mock-(open bars) and BDV-infected (solid bars) rats. Note a significant increase in the number of activated microglia cells as early as 10 dpi. * – p < 0.05 vs. mock-infected animals. (E) The quantitative estimate of the number of granule cells in the dentate gyrus of the hippocampus in mock-(open bars) and BDV-infected (solid bars) rats. Note that a significant decline in the number of granule cells was not observed until 30 dpi. * – p < 0.05 vs. mock-infected animals. (F) The quantitative estimate of the volume of the dentate gyrus of the hippocampus in mock-(open bars) and BDV-infected (solid bars) rats. Note that a significant decrease in the volume of the dentate gyrus of the hippocampus was not seen until 30 dpi. * – p < 0.05 vs. mock-infected animals.

Our pilot experiments had shown that CD11b+ and Iba1+ cells were found in both BDV-infected rats and control animals, indicating that those markers were likely to label both activated and inactivated microglia in rats (data not shown). In contrast, ED1+ microglia were observed predominantly in BDV-infected brains (Fig. 1A and 1B), suggesting that ED1 could be used as a reliable marker for BDV-activated microglia. In order to rule out the effects of inoculation-associated tissue damage on ED+ staining, we had also performed a preliminary comparison of numbers of ED1+ cells in the left (the place of inoculation) and the right (intact) hemispheres and showed no differences between hemispheres at 8, 10 and 17 dpi (data not shown). In addition, compared to intracerebral inoculation, intranasal instillation with the virus produced a similar increase in ED+ cells in the hippocampus (data not shown). Collectively, our preliminary experiments had indicated that ED1 is a specific marker of BDV-induced microglia activation. Thus, ED1-positive staining was used in the study to quantitatively evaluate microglia activation in BDV-infected rats.

Images of brain sections were viewed on fluorescent microscope Nikon Eclipse E400 equipped with the digital camera CoolSnap™ ES (Roper Scientific Production). Estimation of the numbers of ED1+ microglia was performed by counting ED-labeled cells, using microscopic fields sampled in the equidistant parts of the DG in the right (intact from intracranial inoculation) hemisphere as presented on Figure 1B. The average number of ED1+ cells for three adjacent brain sections at the same levels was determined for each rat. Then, the averaged number of ED1+ microglia cells in the DG was determined for each group, with the number of animals per each group being n = 3 (8 dpi); 8 (10 dpi); 5 (17 dpi); 4 (30 dpi). The data are presented as the mean (for each group) ± S.E.M.

For an analysis of neuronal loss in the DG, a stereology-based approach was employed as described before [10]. Briefly, the brains used for ED1 staining were used for the stereology counts as well. Every fifth brain section of one hemisphere from each rat was separately collected in PBS in 12-well plates and stained with cresyl violet. The dimensions of the optical dissector for DG neurons were: height 12.00 μm; guard height 2 μm, spacing 1250 μm. The co-efficient of error (CE) was below 10% for all animals. To count neurons, an image of counting regions was displayed on the video monitor and a square counting box superimposed on the image using the stereology software Stereologer (SPA Alexandria, VA, USA). Sections were viewed with a 100× objective (final magnification on the computer screen: 2200×). The brain hemispheres used for counting neurons were also used for measuring the volumes of the DG, using Cavalieri point counting [10]. The data was analyzed with Student t-test for each time point separately. P value below 0.05 was used as the indication of significant differences.

We found a significant increase in the number of activated microglia cells as early as 10 dpi (Fig. 1B and 1D). The number of ED1+ cells in the brain remained significantly higher in BDV-infected rats until 30 dpi (Fig. 1D). BDV-infected neurons were predominantly found in the areas with the high density of ED1+ cells (Fig 1C). BDV infection during the first three weeks post inoculation was found to be limited to neurons only, with occasional staining of astrocytes being found at 30 dpi (data not shown). Despite this rise in the number of activated microglia cells across the first three weeks after inoculation with the virus, the numbers of granule cells and the volumes of the DG continued to increase at a comparable rate with mock-infected rats until 17 dpi. (Fig. 1E and 1F). It is at the last time point only (i.e., 30 dpi), when a significant decrease in the number of granule cells and the volumes of DG was found in BDV-infected rats compared to control animals (Fig. 1E and 1F). We also evaluated activation of microglia in the CA3 and CA1 areas of the hippocampus where minimal if any neuronal damage was reported [10]. At 10 dpi, BDV infection was associated with significantly more CD1-positive cells: BDV – 25.3 ± 6.4 vs. Mock – 0.7 ± 0.3 in the CA1 area; and BDV – 12.7 ± 5.1 vs. Mock – 1.7 ± 0.5 in the CA3 area (n = 4 for each group, t-test, p < 0.05).

We demonstrated for the first time that neonatal BDV infection activates microglia at least two weeks prior to an appreciable neuronal loss in the DG of the hippocampus, the brain region that undergoes most profound and accelerated neurodegenerative changes [3,7,8]. This data is further supported by the results on a significant activation of microglia in the CA areas of the hippocampus that has been shown to harbor highest amounts of BDV in the absence of detectable signs of neuronal injury [4,10]. The findings show that activation of microglia is an early response to neonatal BDV infection in rats, suggesting that microgliosis could trigger demise of infected neurons or exacerbate on-going subtle dysfunction initiated by the direct virus infection [12-14]. BDV-produced neurodegeneration and perhaps slower adult neurogenesis [15] in the DG will also likely affect the overall composition, average age and identity of DG cells, and will probably have considerable consequences for the connectivity, input and properties of the hippocampal DG-CA3 neuronal circuit, resulting in emotional and cognitive abnormalities observed in BDV-infected rats [5,7].

The present data are consistent with our previous *in vitro *studies that BDV infection of primary neuronal and astrocyte cultures does not induce neuronal toxicity for weeks post infection but increases the number of activated microglia cells in mixed neuronal-astroglia-microglia cultures [16]. The mechanisms whereby persistent non-lytic BDV infection activates microglia remain obscure. Our previous report has demonstrated BDV does not infect microglia *in vitro *and BDV-infected neurons do not activate microglia. It is in BDV-infected mixed cultures containing neurons, astrocytes and microglia that we see activation of microglia in the absence overt neuronal toxicity, suggesting that microglia activation appears a result of complex interactions between BDV-infected neurons, astrocytes and microglia. In a broader context, activation of microglia by neonatal BDV infection resembles several neurodegenerative conditions where microglia may initially respond to subtle abnormalities well before overt neuronal damage is produced [2,17].

Taken together, the present findings provide for the first time direct evidence that neonatal BDV infection produces microglia activation prior neuronal death and suggest that chronic neuroinflammation may contribute to neuronal damage associated with a persistent neurotropic virus infection.

## Abbreviations

Borna Disease Virus: BDV; dentate gyrus: DG; days post infection: dpi; phosphate buffered saline: PBS; standard error of mean: SEM; co-efficient of error: CE.

## Competing interests

The authors declare that they have no competing interests.

## Authors' contributions

MVO and MVP conceived and designed the study. MVO, KM and KS performed the experiments. MWV helped with the stereology analysis. MVO and MVP wrote the manuscript. All authors have read and approved the final manuscript.

## References

[B1] Streit WJ (2002). Microglia as neuroprotective, immunocompetent cells of the CNS. Glia.

[B2] Streit WJ, Mrak RE, Griffin WST (2004). Microglia and neuroinflammation: a pathological perspective. Journal of Neuroinflammation.

[B3] Carbone KM, Park SW, Rubin SA, Waltrip W, Vogelsang GB (1991). Borna disease: association with a maturation defect in the cellular immune response. J Virol.

[B4] de la Torre JC (2002). Bornavirus and the brain. J Infect Dis.

[B5] Pletnikov MV, Moran TH, Carbone KM (2002). Borna disease virus infection of the neonatal rat: developmental brain injury model of autism spectrum disorders. Front Biosci.

[B6] Gonzalez-Dunia D, Watanabe M, Syan S, Mallory M, Masliah E, de La Torre JC (2000). Synaptic pathology in Borna disease virus persistent infection. J Virol.

[B7] Hornig M, Weissenbock H, Horscroft N, Lipkin WI (1999). An infection-based model of neurodevelopmental damage. Proc Natl Acad Sci USA.

[B8] Zocher M, Czub S, Schulte-Monting J, de La Torre JC, Sauder C (2000). Alterations in neurotrophin and neurotrophin receptor gene expression patterns in the rat central nervous system following perinatal Borna disease virus infection. J Neurovirol.

[B9] Weissenbock H, Hornig M, Hickey WF, Lipkin WI (2000). Microglial activation and neuronal apoptosis in Bornavirus infected neonatal Lewis rats. Brain Pathol.

[B10] Dietz D, Vogel M, Rubin S, Moran T, Carbone K, Pletnikov M (2004). Developmental alterations in serotoninergic neurotransmission in Borna disease virus (BDV)-infected rats: a multidisciplinary analysis. J Neurovirol.

[B11] Haas B, Becht H, Rott R (1986). Purification and properties of an intranuclear virus-specific antigen from tissue infected with Borna disease virus. J Gen Virol.

[B12] Gonzalez-Dunia D, Volmer R, Mayer D, Schwemmle M (2005). Borna disease virus interference with neuronal plasticity. Virus Res.

[B13] Hans A, Bajramovic JJ, Syan S, Perret E, Dunia I, Brahic M, Gonzalez-Dunia D (2004). Persistent, noncytolytic infection of neurons by Borna disease virus interferes with ERK 1/2 signaling and abrogates BDNF-induced synaptogenesis. FASEB J.

[B14] Volmer R, Prat CM, Le Masson G, Garenne A, Gonzalez-Dunia D (2007). Borna disease virus infection impairs synaptic plasticity. J Virol.

[B15] Solbrig MV, Adrian R, Baratta J, Lauterborn JC, Koob GF (2006). Kappa opioid control of seizures produced by a virus in an animal model. Brain.

[B16] Ovanesov MV, Sauder C, Rubin SA, Richt J, Nath A, Carbone KM, Pletnikov MV (2006). Activation of microglia by borna disease virus infection: in vitro study. J Virol.

[B17] Yoshiyama Y, Higuchi M, Zhang B, Huang SM, Iwata N, Saido TC, Maeda J, Suhara T, Trojanowski JQ, Lee VM (2007). Synapse loss and microglial activation precede tangles in a P301S tauopathy mouse model. Neuron.

